# Vulnerability to bending and occlusion of distal lumen of the 17G triple-lumen central venous catheter

**DOI:** 10.1186/s40981-024-00691-7

**Published:** 2024-02-03

**Authors:** Tomohiro Yamamoto

**Affiliations:** Division of Anesthesiology, Niigata University Graduate School of Medical and Dental Sciences, 1-757, Asahimachi-Dori, Chuo Ward, Niigata, 951-8510 Japan

To the Editor

The risk of central venous catheter (CVC)-related venous thrombosis [1, 2] should always be kept in mind. It is a critical issue, particularly in pediatric patients with congenital heart diseases, because of the possible impact on subsequent surgical treatment. The risk of CVC-related venous thrombosis is reportedly greater when CVC is larger than one-third the diameter of the target vein [3]. Cardinal Health™ has recently developed a 17-gauge (G) triple-lumen CVC (ARGYLE™ Fukuroi SMAC^TM^ Plus), of which the cross-sectional area of lumens and flow rate are comparable to those of a 15G triple-lumen CVC (Fig. 1).

**Fig. 1:**
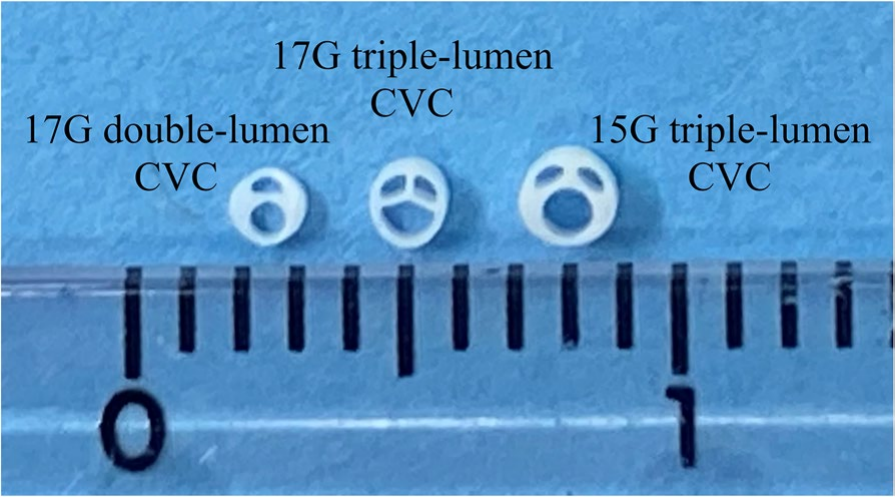
Cross-section of central venous catheters (CVCs). Cross section of the 17-gauge (G) double-lumen (left), 17G triple-lumen (center), and 15G triple-lumen (right) CVCs (ARGYLE™ Fukuroi SMAC™ Plus, Cardinal Health™), side by side with ruler scale for size comparison. Note the semicircular-shaped distal (largest) lumen of the 17G triple-lumen CVC (center) in contrast with the circular-shaped distal lumens of the 17G double-lumen (left) and of the 15G triple-lumen (right) CVCs. The outer diameters of the 17G double-lumen (left), 17G triple-lumen (center), and 15G double-lumen CVC (right) are 1.35 mm, 1.45 mm, and 1.70 mm, respectively

The distal lumen is generally thought to be the least susceptible to catheter bending and the least vulnerable to occlusion because it has the largest lumen and is located in the middle of the catheter. However, uncommonly, we have encountered several cases in our hospital wherein the pressure alarm sounded on the syringe pump connected to the distal lumen or no blood backflow was obtained from the distal lumen of the 17G triple-lumen CVC, whereas the other two smaller lumens had no problems. We found that the CVCs were bent at the site just distal to the junction hub (Fig. 2), which was common in all catheter obstruction cases. However, 17G double-lumen CVCs had been used in our hospital without similar problems. The cross-sectional structure of the CVCs is shown in Fig. 1, where the lumens of the 17G triple-lumen CVC have a semicircular or fan-shaped structure. Additionally, the distal lumen, which has a semicircular structure, is the most vulnerable to bending, while the lumens of the 17G double-lumen and 15G triple-lumen CVCs have a circular or crescent-shaped structure. To ensure a large lumen despite the smaller outer diameter of the 17G triple-lumen CVC, the catheter structure and septal walls between the lumens were thinner than those of the 17G double-lumen or 15G triple-lumen CVCs (Fig. 1).

**Fig. 2:**
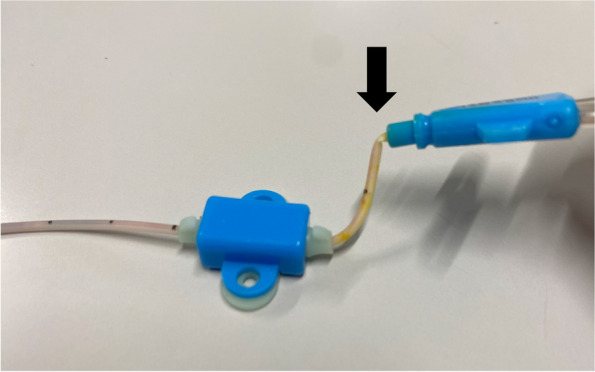
A 17G triple-lumen CVC with an obstructed distal lumen. A 17G triple-lumen CVC after removal from one of the cases of a distal lumen occlusion problem. Note an acute bend at the site just distal to the junction hub (black arrow), which is observed in all catheters with obstructed distal lumen

Figure 3 shows a method to reinforce the 17G triple-lumen CVC prior to CVC insertion procedure. As an example (Fig. 3a), an extension tube for the SAFE ACCESS™ (Cardinal Health™) infusion set is employed. After calculating the CVC insertion depth as previously described [4], the extension tube for the SAFE ACCESS™ (Cardinal Health™) infusion set is cut to the appropriate length, and the CVC is passed through it deep enough to cover the weak part of the CVC completely, the site just distal to the junction hub (black arrows in Fig. 3a). Thereafter, the fixture is attached. Some anesthesiologists in our hospital use a 16 Fr suction tube (ARGYLE™ Fukuroi, Cardinal Health™) to completely cover the junction hub (black arrows) to the rubber part of the fixture (white arrows) (Fig. 3b). The CVC bending and occlusion problem has been completely resolved in our hospital since this method was introduced. This method can be performed using inexpensive items found at any facility, such as intravenous lines or suction tubes. It is helpful for protecting patients from the risk of circulatory instability caused by catecholamine dosage instability.

**Fig. 3:**
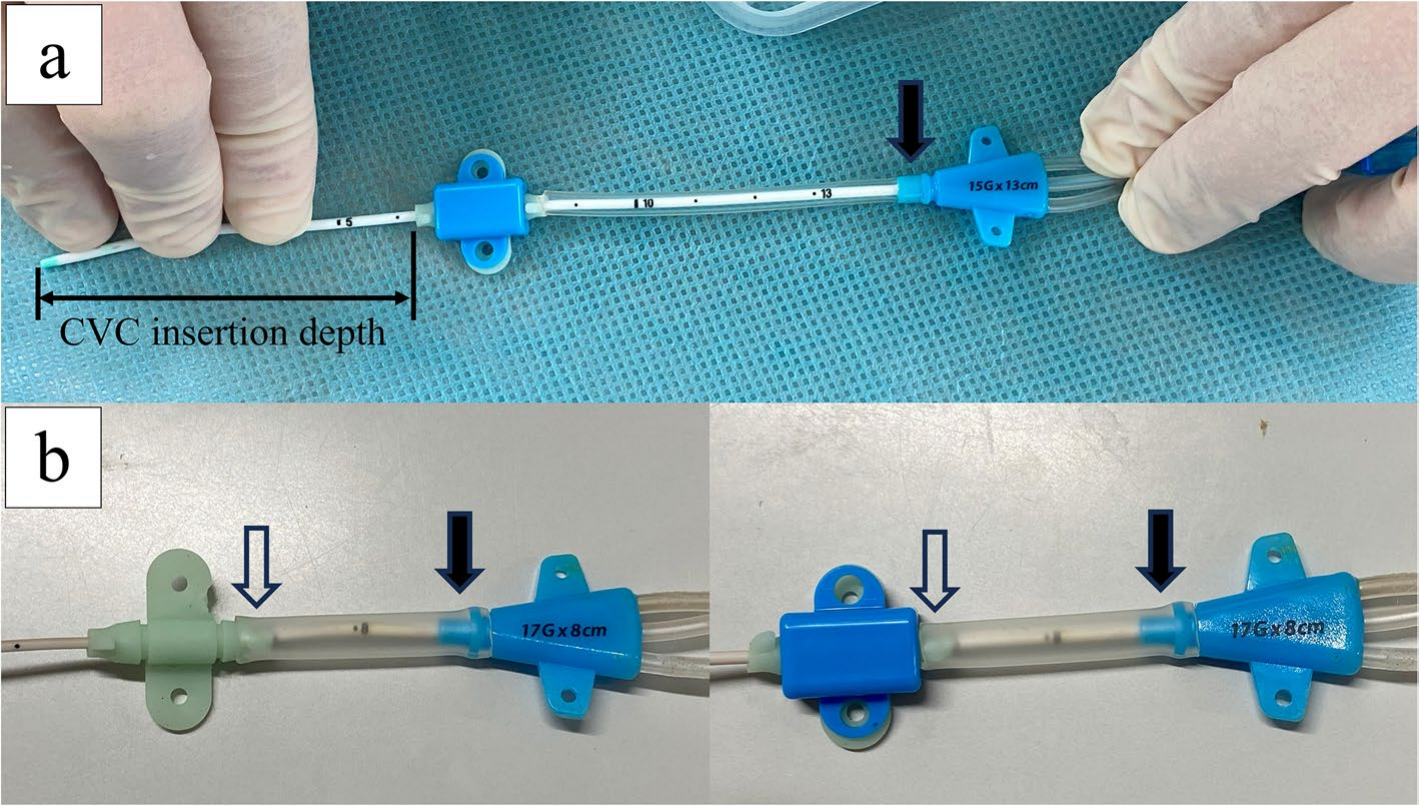
Reinforced 17G triple-lumen CVCs prior to insertion procedure. **a** An extension tube for the SAFE ACCESS™ (Cardinal Health.™) infusion set and **b** a 16 Fr suction tube (ARGYLE^TM^ Fukuroi, Cardinal Health^TM^), covering the 17G triple-lumen CVC from the junction hub (black arrows) to the rubber part of the fixture (white arrows)

## Data Availability

The data in this paper are available from the corresponding author upon reasonable request.

## Abbreviation

CVC: Central venous catheter
